# Conventional clinical and prognostic variables in 150 oral squamous cell carcinoma cases from the indigenous population of Karachi

**DOI:** 10.12669/pjms.323.9905

**Published:** 2016

**Authors:** Muhammad Mohiuddin Alamgir, Qamar Jamal, Talat Mirza

**Affiliations:** 1 Prof. Dr. Muhammad Mohiuddin Alamgir, MBBS, MPhil. Department of Pathology, Bahria University Medical & Dental College, Karachi – Pakistan; 2 Prof. Dr. Qamar Jamal, MBBS, MPhil, PhD. Department of Pathology, Ziauddin University Clifton Campus, Karachi – Pakistan; 3 Prof. Dr. Talat Mirza, MBBS, MPhil, PhD. Department of Pathology, Dow University of Health Sciences, Karachi – Pakistan

**Keywords:** Oral squamous cell carcinoma, Clinico-pathological parameters, Clinical stage

## Abstract

**Objective:**

To analyze clinical and prognostic variables of Oral Squamous Cell Carcinoma (OSCC) cases from the indigenous population of Karachi and to correlate with the common risk factor of tobacco habit.

**Methods:**

The study was conducted at Ziauddin University, Karachi. One hundred fifty OSCC cases were collected from the Oncology Department of Ziauddin University Hospital, North Nazimabad, Karachi and Otolaryngology ward of Civil Hospital, Karachi, during 2011 and 2015. The reporting included demographic details and variables like intra-oral subsites, clinical stage and histological grade. Recurrence of tumor after initial resection was also documented.

**Results:**

The patient’s population comprised of 98 males and 52 females. The mean age was 47.1± 12.22 (range:20-78 years). Maximum numbers were seen in the 41–50 years age group. Urdu-speaking community was the most affected ethnic group (n=75). Clinico-pathological analysis revealed that majority of cases were moderately differentiated (59%) and were either clinical stage II (35%) or IV (29%) tumors. The most common intra-oral subsite came out to be buccal mucosa of cheeks (56%) followed by lateral borders of tongue (21%), lips (13%), alveolar (6%), palate (2.6%) floor of mouth (1.3%), etc. Recurrence was observed in 08 out of 150 cases. All patients underwent primary resection±neck dissection and reconstruction where possible.

**Conclusions:**

Overall experience with oral squamous cell carcinoma shows that it has a high tendency for local invasion as well as dissemination to regional lymph nodes, i.e. cervical lymph nodes, both are associated with a poor prognosis. Preventable risk factor of tobacco chewing has been observed in majority of these cases.

## INTRODUCTION

Oral cancers are the eight most frequently occurring cancer worldwide and around 90% are squamous cell carcinomas.^1^ Oral squamous cell carcinoma (OSCC) contributes to the global cancer burden with an estimated 275,000 new cases each year.^2^ It ranks first or second among tumors prevalent in countries like India, Pakistan, Afghanistan, Sri Lanka, Bhutan, Nepal, Iran, and Maldives.^3^ According to the Pakistan Medical Research Council (PMRC) data, in its first report, oral cancer was the commonest cancer among males and second highest to breast in females while in the second report it was the second commonest malignancy in both genders.^4^ Published data by Karachi Cancer Registry (KCR) for Karachi south puts OSCC as the second most common cancer in both genders and the incidence documented therein is the highest reported worldwide.^5^

It is unfortunate that despite of easy approachability of this tumor the overall survival of patients with OSCC has not improved significantly, with 5-year survival rate ranging between 45-50%.^6^ The quality of life for these patients has only marginally improved over the past few decades. Variables historically used to predict prognosis of OSCC include: TNM stage, histopathological grade and to some extent the intra-oral subsite of the lesion.

The outcome of oral cancers has been greatly influenced by the clinical stage at the time of diagnosis. For instance in case of mobile tongue, survival rates range from 80% for individuals diagnosed with stage I lesions to only 15% with those presenting with stage IV disease.^7^ The widely employed Tumor-Node-Metastases (TNM) staging system describes the anatomic extent of the primary tumor as well as the involvement of regional lymph nodes and distant metastasis.^8^ It is supplemented by variables such as the site of occurrence within the oral cavity and the status of surgical margins. For histological grading the WHO grading system recommends three categories: well differentiated, moderately differentiated and poorly differentiated.^9^ This usually depends on the subjective assessment of the degree of keratinisation, cellular and nuclear pleomorphism, and mitotic activity.^10^ It is widely accepted that prognosis is better in early grade cancers, particularly those that are well-differentiated.^11^

OSCC may arise in any part of oral cavity. The site of occurrence shows geographical variation reflecting exposure to varying risk factors in different parts of the world. Among European and American populations tongue is the most frequent site amounting to 40-50% of all oral cancers. Among Asians buccal cancer is more common.^2^ Small symptomless tumors are reported to be most frequent in the floor of mouth, ventero-lateral tongue and soft palate complex.^12^

### Rationale

Several studies have been carried out in both India and the Western countries on risk factor association with clinic-prognostic aspects of Oral cancer. Through these studies researchers have tried to uncover the pathways of carcinogenesis for this lethal cancer. Pakistan has one of the highest reported incidences of oral cancer. In this scenario analysis of tumor characteristics and their association with the common risk factor in the indigenous population of Karachi becomes very relevant.

## METHODS

This study was carried out on 150 cases of clinically diagnosed, histopathologically proven OSCC cases from two tertiary-care hospitals of the city catering these advanced-stage oral cancer cases. One being a public sector Civil Hospital, Karachi and the other was a private sector Ziauddin University Hospital, North Nazimabad, Karachi. Patient enrollment started in Jan 2011 and continued until October 2015. The study was registered at the Ziauddin University, Karachi. After obtaining informed consent from all participants in the study, personal details were recorded on a proforma upon interview. Information regarding age, gender, ethnicity, tobacco-habit, tumor location within the oral cavity, its size, nodal status, histologic grade and clinical stage were recorded. Patients with cancer of any site other than oral cavity or those who did not give consent for research were not included in the study. All cancers were re-confirmed by review of histopathology to be squamous cell carcinoma.

### Inclusion criteria

Presence of histologically proven SCC of oral cavity, age above 10 years and those who voluntarily consented for research.

### Exclusion criteria

Cancer of any site other than oral cavity or any other serious disease, age 10 years or below and those who did not give consent for research. The study protocol was approved by the Ziauddin University Ethics Committee (ZU ERC) for human research and Institutional Review Board (IRB), Dow University of Health Sciences, Karachi.

## RESULTS

**Fig.1:**
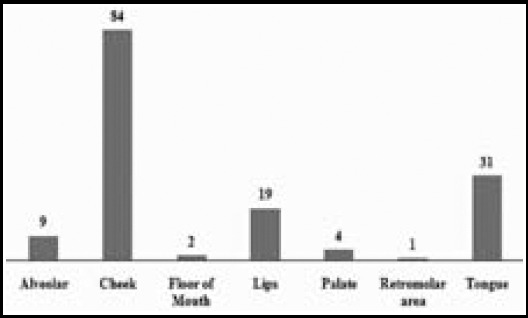
Distribution of OSCC cases according to intra-oral subsite of the lesion.

A total of 150 OSCC cases were registered. 98 patients were males and 52 were females, male/female ratio being 1.88:1. The age of patients ranged from 20-78 years (mean 47.1±12.22) and the most commonly involved age bracket was between 41 to 50 years (30.6% of all cases). Cheek (buccal mucosa) was the most common site involved in both sexes (n=84, 56%) followed by lateral borders of tongue (n=31, 21%), lips (n=19, 13%), alveolar mucosa (n=9, 6%), palate (n=4, 2.6%) and floor of mouth (n=2, (1.3%). (Table-I, Fig.1).

**Table-I:** Distribution of 150 OSCC cases according to age, gender & site of lesion.

*Age Groups(Years)*	*Intra-oral subsite of the lesion*														*Total*

	*1*		*2*		*3*		*4*		*5*		*6*		*7*		

	*M*	*F*	*M*	*F*	*M*	*F*	*M*	*F*	*M*	*F*	*M*	*F*	*M*	*F*	
11-20			1											1	2
21-30		1	6				3		1				2	1	14
31-40	1		20	1			3	1					5	4	35
41-50	1	1	18	11			2	4		1			4	4	46
51-60	5		10	9		1	2	1	1		1		4	2	36
61-70			3	3	1			3	1				2	1	14
71-80			1	1										1	3
Total	7	2	59	25	1	1	10	9	3	1	1	0	17	14	150

*Key:* 1-Alveolar, 2- Cheek, 3- Floor of mouth, 4- Lips, 5- Palate, 6-Retromolar area, 7-Tongue.

Most of the cases belonged to the Urdu-speaking community (n=75, 50%). Others include Sindhi (n=20, 13.3%), Memon/Baloch (n=17 each, 11.3%), Punjabi/Pathans (n=8/7, approx. 5% each) and others (n=6, 4%).

Assessment of tobacco habits revealed exclusive tobacco chewers consuming smokeless tobacco in forms like Gutka, Paan, Manpuri, Niswar and Mawa etc., to be present in more than half of patients (58%). Mixed tobacco habit of chewing plus smoking was seen in 20% cases. 17% patients gave no history of tobacco use while only 5% were exclusive smokers.

**Fig.2:**
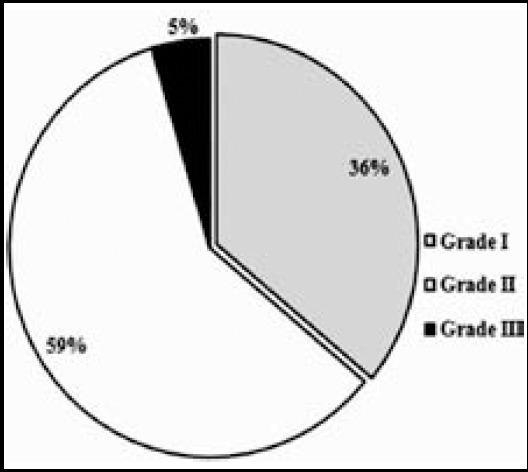
OSCC percentages according to histological grade of tumor.

Analysis of histological grading showed grade II moderately differentiated OSCC in more than half (59%) of patients. Grade I well differentiated tumors were 36%, while grade III poorly differentiated tumors were only 5% of cases (Fig.2).

**Fig.3:**
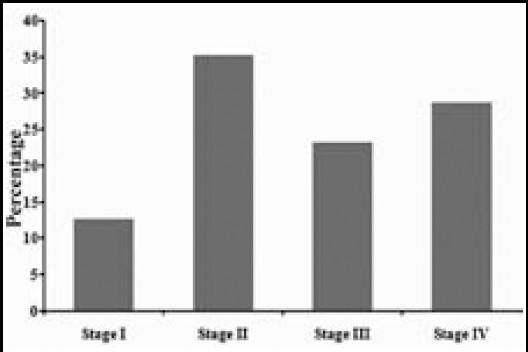
OSCC percentages according to the clinical stage of tumor.

TNM staging revealed that majority of cases were in either clinical stage II /35% or clinical stage IV/29% (Fig.3). Recurrence at the primary site or regional lymph nodes was seen in 08 cases, most belonging to either stage III or IV.

## DISCUSSION

Most of the studies done worldwide reported about 90% of oral cancers in individuals over 40 years of age with 65 as the average age at diagnosis.^13^ However, several other investigators have reported an increase in oral cancer diagnosed in younger individuals.^14^ A multicentre study conducted in Pakistan during 1973-74 showed that the incidence was very small in the initial age groups but from 45 years onwards it started rising, peaks around 60-64 years and declined afterwards.^15^ Mirza et al.^16^ during late 90s reported maximum number of cases in 51 to 60 years age group. In our study we found maximum number of cases in the 41 to 60 years age bracket. However, a substantial number was also noted in the younger age group of 31-40 years. This may be attributable to the trend of early exposure to smokeless tobacco with additives in our population.

For decades oral cancer has been affecting males more often than females, however, in the past one decade or so the ratio has narrowed down to two men for each women. Nearly a quarter of the newly diagnosed cancers in males from Sri Lanka, India, Pakistan and Bangladesh are located in the head and neck region.^17^ Our study showed a similar trend with a male to female ratio of 1.88:1.

From Karachi city Akram S et al.^18^ reported involvement of cheek mucosa to be 54% followed by tongue 24%, alveolus 9%, lip 7% and floor of the mouth 6%. More recently Zafar M et al.^19^ also reported cheek mucosa as the commonest site (61.4%) followed by tongue (32.1%). In our study, the overwhelming majority of patients suffered from cheek cancer followed by that of tongue and lips. Here again the gene-environment interaction being the major player as the chewable forms of tobacco are kept in the buccal pouch for long periods in our study population for a maximum chewing experience. This results in greater exposure of the subjected mucosa to carcinogens.

Tumor histological grade is one variable which is strongly associated with behavior of any malignant tumor as regards to aggressiveness and spread. A tertiary-care hospital from Karachi reported 59.53%, 32.55% and 7.9% grade I, II and III OSCC cases, respectively.^20^ Akram S et al.^18^ reported 60% grade I, 36% grade II and 4% grade III tumors in 100 OSCC cases. While Zafar M et al.^19^ reported 25% grade I, 55% grade II and 20% grade III tumors in 140 OSCC cases. Our study endorses the last report as we found grade II tumors as most prevalent (59%) followed by grade I (36%). Only 5% of our cases were the most anaplastic grade III tumors.

In early stage cancers treatment is less complicated and prognosis is better.^21^ A study from Karachi conducted in the 70s found stage III/IV tumors as 44%/26% of all studied cases, respectively.^22^ A similar study in the 90s endorsed this observation while reporting stage III/IV cancers to be 34.6% and 21.5%.^23^ Zafar M. et al.^19^ in 2015 reported 44.2% stage I/II and 55.7% stage III/IV tumors out of 140 OSCC cases from Karachi. In our study clinical stage III and IV make out 52% of all cases while in contrast to other studies stage II came out to be the largest group with 35% of all cases. This may partly be explained by the lack of proper facilities required for correct clinical staging, especially reliable scanning reports, available to the treating oncologist or surgeon. In addition we also found deficiencies in proper documentation of the extent of disease at the time of surgery.

## CONCLUSION

A changing trend towards almost equal involvement of younger as well as middle-aged individuals was obvious. OSCC has a high tendency to spread and hence to present with advanced stage. There is a need for more meticulous clinical staging so as to select best treatment modality as well as to predict prognosis in these advanced stage tumors. Finally, the presence of the devastating effect of chewable tobacco as a risk factor for OSCC emphasizes the need for primary prevention.
